# Lack of *TEK* Gene Mutation in Patients with Cutaneomucosal Venous Malformations from the North-Western Region of Algeria

**DOI:** 10.1155/2013/784789

**Published:** 2013-12-09

**Authors:** Nabila Brahami, Mourad Aribi, Badr-Eddine Sari, Philippe Khau Van Kien, Isabelle Touitou, Gérard Lefranc, Mouna Barat-Houari

**Affiliations:** ^1^Laboratory of Applied Molecular Biology and Immunology, University of Tlemcen, 13000 Tlemcen, Algeria; ^2^Service de Stomatologie et de Chirurgie Buccale du Centre Hospitalier et Universitaire de Tlemcen, 13000 Tlemcen, Algeria; ^3^Génétique Médicale, Laboratoire de Cytologie Clinique et Cytogénétique, CHU de Nîmes, Place du Professeur Robert Debré, 30029 Nimes Cedex 9, France; ^4^Unité Médicale des Maladies Auto-Inflammatoires, Département de Génétique, CHRU, Montpellier, 34961 Montpellier Cedex 2, France; ^5^Université Montpellier 1, 34961 Montpellier Cedex 2, France; ^6^Génétique des Maladies Auto-Inflammatoires et des Ostéo-Arthropathies Chroniques, INSERM U844, 34091 Montpellier Cedex 5, France; ^7^Laboratoire d'Immunogénétique Moléculaire, Institut de Génétique Humaine, CNRS UPR 1142, et Université Montpellier 2, 34095 Montpellier Cedex 5, France

## Abstract

*Background*. Venous malformations (VM) result from an error in vascular morphogenesis. The first gene suspected in their development is the *TEK* gene (tyrosine kinase, endothelial). Mutations of this gene have been identified in several Belgian families with a dominant form of the disease. Therefore, we investigated whether mutations in this *TEK* gene could explain the MV development in patients of families from Tlemcen region (north-western Algeria). *Methods*. Genomic DNA was extracted from leucocytes of ten patients. The search for mutations in all the 23 exons and in the 5′ and 3′ intronic sequences flanking the *TEK* gene was performed using PCR amplification and direct sequencing of amplified genomic DNA. Additionally, a search for somatic mutations of the gene *TEK* was performed on a biopsy of the venous malformation from one of the ten eligible patients. *Results*. The sequencing of the 23 exons of the *TEK* gene revealed neither germinal mutation in our ten patients nor somatic mutation in the tissue of the biopsy. *Conclusion*. The absence of mutation in the *TEK* gene in the population studied suggests that the *TEK* gene is not necessarily involved in the onset of VM; its association with these malformations may differ from one population to another.

## 1. Introduction

Vascular malformations (VM) are benign vascular lesions that are described as structural congenital anomalies [1]. These lesions are always present at birth, but may not be visible until days, weeks, or even years after birth [2]. VM are classified according to the type of involved vessels, such as arterial, venous, lymphatic, capillary malformations, or a combination of different vessels [1–3].

VM result from an error in vascular morphogenesis [4]. They are present at birth and their growth is usually gradual and steady during the first year of life [3]. Their evolutionary peak is generally observed in adolescence or after a traumatic event [5]. Localized facial forms are present essentially on the lips, eyelids, and tongue [5, 6]. The extension of the gingival mucosa can result in bleeding, which can occur spontaneously and/or after certain dental procedures and treatments [7].

Some locations cause, by mass effect, skeletal deformities in the frontoorbital region with enophthalmos and maxillomandibular and dentoalveolar defects, with repercussions on the dental bite and open bite [5, 8]. An important lip deformity tends to make them incompetent. Likewise, giant VM of the tongue can cause permanent changes in normal dental occlusion [7, 8]. Additionally, superficial VM can lead to disfigurement, resulting in an asymmetry hardly supported by patients [9, 10]. This condition is often the cause of low self-esteem and school failure in children and adolescents.

Although the real causes of the development of VM are still poorly understood, previous studies have suggested the involvement of genetic factors in their occurrence [11]. First, the *TEK* gene (also called *TIE*-*2*, tyrosine kinase with immunoglobulin and epidermal growth factor homology domain 2), located at chromosomal region 9p21 and encoding the endothelial-specific receptor tyrosine kinase, has been implicated in Belgian families with a dominant form of the disease [11, 12].

To date, seven missense mutations were identified in these Belgian patients with dominant transmission, including 2545C>T in exon 15, 2690A>G, 2744G>A, 2752C>T, 2755G>T, and 2773G>T in exon 17, and 3300G>C in exon 22 [11, 13, 14]. These mutations lead to an excess of angiopoietin 1 signaling, a ligand for TIE-2 receptor, and consequently to an abnormal proliferation of endothelial cells [11].

The relatively high frequency of VM in Tlemcen region (north-western Algeria) compared to others [15] led us to search in all exons of the *TEK* gene for either already described mutations or other not yet identified.

## 2. Materials and Methods

### 2.1. Patients and Families

**Figure 1:**
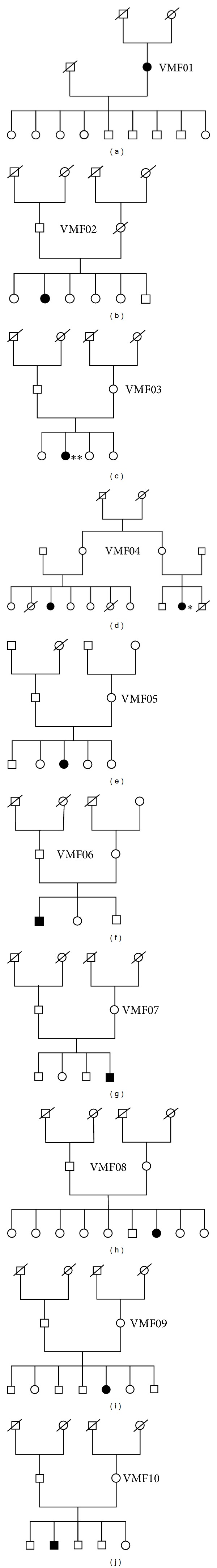
Genealogical trees of ten Algerian families analyzed for *TEK* gene. *Patient VMF04.III.3 not included in the molecular study. **For patient VMF03.III.2, the DNA in the tissue of the biopsy was sequenced.

A total of ten (10) families, each with a patient with VM (3 men and 7 women), were recruited in Stomatology and Oral Surgery Department of Tlemcen University Medical Centre (Figure 1).

**Figure 2:**
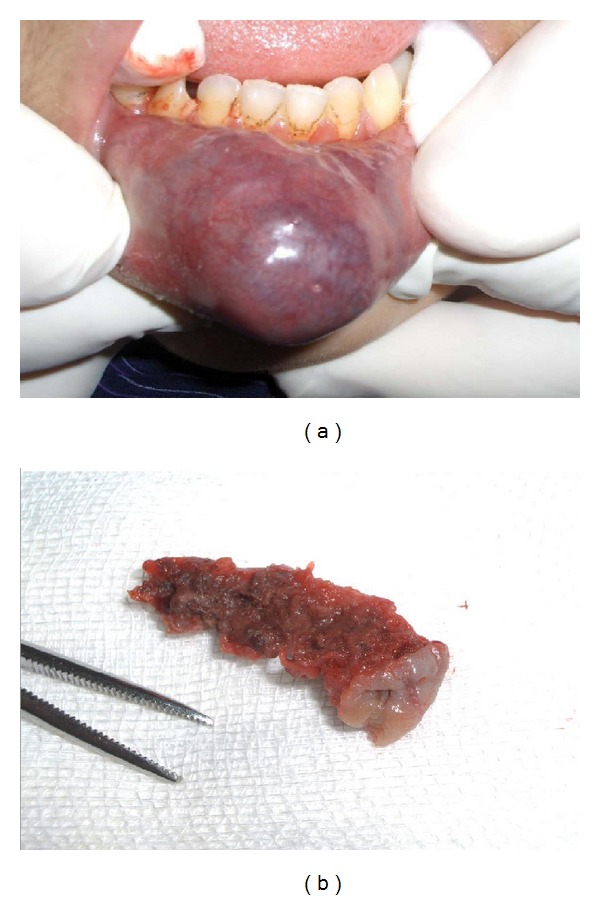
Aspect of the labial venous malformation seen after inclined position and surgical fragment collected following resection using multiple times of transmucosal embolization.

The characteristics of the patients and family members were recorded using a detailed questionnaire. The mean age (± standard error) of the patients at diagnosis was 12 ± 1 years. The recruitment of the patients was carried out on the basis of clinical examination. Such malformations are characterized by a soft, compressible, nonpulsatile tissue mass that does swell in inclined or proclive position [6, 16] (Figure 2).

**Figure 3:**
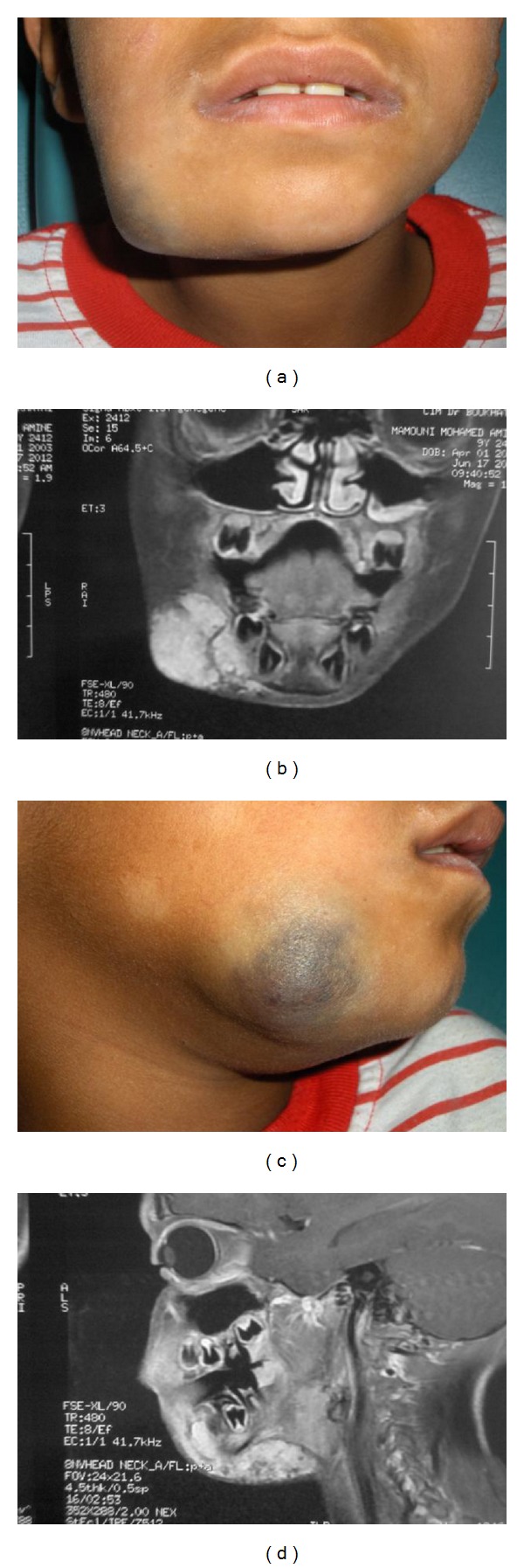
A magnetic resonance imaging (MRI) of superficial and deep venous malformation. The technique was performed in three planes with T2 fat-saturation (T2-FS), axial T1, 2D time-of-flight (2D TOF), magnetic resonance angiography (MRA) of supra-arterial trunks, and three planes T1-FS for the cervical region.

A magnetic resonance imaging (MRI) was performed in the case of malformations that are simultaneously superficial and deep (Figure 3). The main inclusion criteria were the geographical location of patients exclusively in the region of Tlemcen and inactive malformations. The exclusion criteria were especially arteriovenous malformations. All participants or their parents or guardians have signed an informed consent in accordance with the latest version of the Helsinki Declaration. This study was approved by the Local Ethics Committee and supported by the Hubert Curien Partnership (PHC Tassili, Code 10MDU794).

### 2.2. Samples

A total of 52 blood samples were collected into EDTA-containing Vacutainer tubes (BD Vacutainer EDTA, USA). VM tissues were taken from one patient after surgery (VMF03.III.2/family VMF03), immediately placed into a sterile collection tube in liquid nitrogen and, then, stored at −80°C in dry ice.

### 2.3. DNA Analysis

For all samples, DNA was extracted from blood cells using QIAamp DNA Blood Midi Kit Qiagen, as recommended by the manufacturer (Qiagen Valencia, CA, USA). The tissue DNA was isolated using spin columns with the QIAamp DNA Blood Mini Kit Qiagen. The DNA concentration was then measured by spectrophotometer NanoDrop ND-1000 at 260 nm and then at 280 nm (NanoDrop Technologies, Wilmington, DE).

The search for mutations in all the 23 exons as well as in the 5′ and 3′ intronic sequences flanking the exons of the *TEK* gene was performed by PCR amplification followed by direct sequencing of amplified DNA segments. Such analyses were performed in the Genetics Laboratory of the Medical Unit for Auto-Inflammatory diseases, Hospital Arnaud de Villeneuve, Montpellier, France.

The primer sequences were specifically established to amplify each exon (Table 1), using the Primer3 program [17] (v.0.4.0), referring to the *TEK* gene sequence (ENSG00000120156) published in Ensembl [18].

The DNA was amplified in a thermocycler for PCR (Applied Biosystems, Foster, CA), using the primers described in Table 1. The medium of the DNA amplification reaction was composed of 50 ng of DNA, 25 *μ*M of each primer, and 2X Promega PCR Master Mix (Promega). The PCR conditions were as follows: 5 minutes at 95°C followed by 35 cycles of 30 seconds of denaturation at 95°C, primer annealing at 60°C for 30 seconds, and elongation at 72°C followed by one cycle at 72°C for 10 minutes.

After checking the quality and size of the PCR products by agarose gel (1.5%) electrophoresis, a bidirectional sequencing was performed. The amplification products were bidirectionally sequenced with Mix BigDye Terminator kit version 3.1 (ABI). The sequences of the 23 exons and their flanking regions were compared with the *TEK* gene reference sequence published in Ensembl [18], using the SeqScape v2.5 software (ABI).

## 3. Results

### 3.1. Phenotype of Patients

Table 2 shows the clinical data of eleven patients with VM, ten of which had a lip malformation and one had a genio-cervical malformation (one of the eleven patients was not included in the molecular study). Eight patients are female; one of the ten families (VMF04) accounts for two patients with venous malformation (Figure 1).

### 3.2. Molecular Analysis of the *TEK* Gene

**Figure 4:**
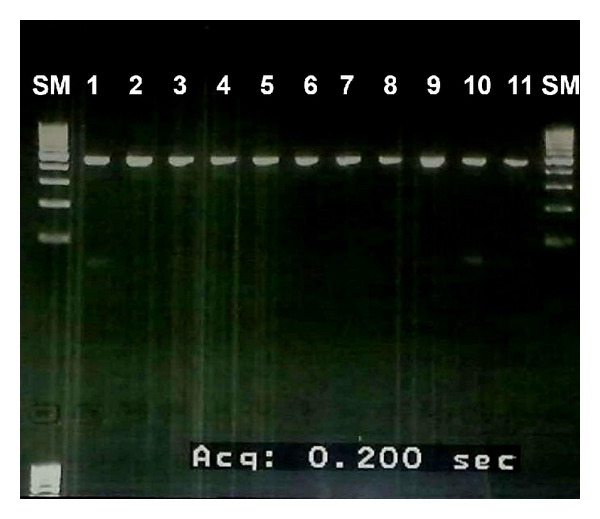
Amplified fragments detected on 1.5% agarose gel electrophoresis of exon 17 for DNA from blood cells (lanes 1 to 10) and tissue of the biopsy (lane 11) of patients with venous malformation. Lane SM: 100 base-pair ladder (size markers).

The amplification fragments were obtained for each of the 23 exons of the *TEK* gene, using DNA from blood samples of ten patients and VM lip tissues from one patient (VMF03.III.2) after surgery.  Figure 4 shows the amplification fragments of exon 17 from genomic DNA of the ten patients included in this study and from somatic DNA of patient VMF03.III.2.

**Figure 5:**
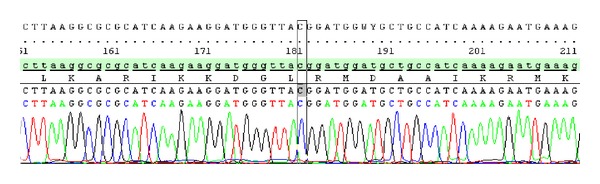
Result of direct sequencing of codon 849 of exon 15 in all the studied patients with venous malformation.

None of the mutations previously described, including even the mutation of the CGG codon 849 (2545C>T) in exon 15, reported by Wouters et al. [13], in six out of twelve Belgian families with a hereditary form of VM, has been detected after ten blood and one VM tissue DNA analyses of all the 23 exons of the *TEK* gene (Figure 5).

## 4. Discussion

In this study, we present the results of the *TEK* gene sequencing in ten unrelated patients from Tlemcen in the north-western region of Algeria with VM.

According to the previous analyses for different mutations in the germline DNA [11, 13, 14], we have to firstly look for these mutations, and, then, we extended analyses to all the 23 exons of the *TEK* gene. Since no germinal mutation was found in all the samples, we searched for somatic mutations in tissues of a VM biopsy.

The lack of mutation suggests that there is a mutated gene other than *TEK* or other factors which would be involved and responsible for VM development in our population.

The vascular endothelial cell (EC) specific receptor tyrosine kinase TEK plays a crucial role in angiogenesis and cardiovascular development [19–27]. Its ligands, the angiopoietins (Ang), induce receptor dimerization and phosphorylation [28], with Ang-1 acting as an agonist and Ang-2 as context-dependent antagonist or weak agonist [29, 30].

Other mutation players within the TEK pathway, especially those proximal to the receptor, could also be expected to yield similar, specific phenotypes. These include TIE-1, which can heterodimerise with TEK [31, 32]; the Ang ligands [29–35]; and the vascular endothelial protein tyrosine phosphatase (VE-PTP), which can specifically regulate *TEK* activation [36, 37].

Ang-1 stimulation of *TEK* has recently been found to induce the expression of Apelin, a ligand for the G-protein-coupled receptor (GPCR, also known as the APJ receptor), in ECs. This system in turn regulates the caliber of blood vessels [38] making it an attractive, as yet untested, candidate pathway through which the dilated channels in VM may occur.

Venous anomalies represent a significant fraction of patients seen at centers specialized in treating VM, as they can cause pain and affect appearance or organ function, due to their size, localization, and expansion.

The majority of VM are sporadic in nature and characterized by extensive, unifocal lesions of variable size, which can infiltrate deep into underlying tissues.

The search for mutations in other candidate genes (*ANG1*, *ANG2*, *TIE1*, *VE-PTP* [vascular endothelial protein tyrosine phosphatase] and the *APLN* genes) in the DNA from biopsies is the next step. If this proves unsuccessful, it will be necessary to achieve exome sequencing analysis, or whole genome sequencing, and to compare the sequences with those of exons and the entire genome of germline DNA from leukocytes of corresponding patient.

## 5. Conclusions

The absence of mutation in the *TEK* gene in the population studied suggests that the *TEK* gene is not necessarily and not the sole gene involved in the onset of VM. It will be interesting to search on other mutated gene(s) responsible for the development of these malformations on much more patients. The exome sequencing should greatly help to unmask the other involved gene(s), which will be validated only after a study of random samples of the Tlemcen population, in order to ensure that it is not a neutral polymorphism, and that the mutation(s) is or are not found in healthy subjects. If so, the knowledge of the molecular and cellular dysfunctions in the signaling pathway that occur in the formation of the vascular network in endothelial cells will be greatly improved. Consequently, better therapeutic approaches to treat the abnormal angiogenesis of these patients could be developed.

## Figures and Tables

**Table 1:** Primer sequences of all the exons of the *TEK* gene.

Exon	Amorce sens (5′-3′)	Amorce antisens (5′-3′)	PCR product
Exon1.1	AGTCTGAGAAGGATTGGTCATCA	CTGTCTGAGCACAGGGAGTTT	333 bp
Exon1.2	CAGCCCTGCTGATACCAAAT	CACTGATGAGATTTGGGGAGA	409 bp
Exon2	GTTTACCCAATGGGGTCATG	AGCAGCTGCCAAGACAAAAG	448 bp
Exon3	AACGCATTAGCCACCACTGT	ACATCTGCCCACAAGACCA	360 bp
Exon4	CTGAATAGTTCAGCATTTTCATTCT	CAATGCCTGGTTTTTGCTTA	422 bp
Exon5	CTCCTTGTCTTTGTTTCTGTCG	AAATTCTAGATCCAGCAACGATG	399 bp
Exon6	GTTCATCCTACCATGCCACA	TGATTCAAAATCCTGTTGTCCA	413 bp
Exon7	AGTTGGCATGATAGGAGCTCA	GGATGGAAACAAAAGAGGCTT	453 bp
Exon8	TCATCCACATCACAGGTGTCT	GTCAGTTCTGCCTCTCCAGG	469 bp
Exon9	TGGGGTCAATGTTATGGACC	TCCTGGAAATTACCCCAAAG	335 bp
Exon10	ATCACAAAACCTCAAAGCCG	AGCCACCACCTTGAGGTAGA	331 bp
Exon11	TTTCAAAAGCCTAATTTTCCTCA	CACCCATTCAAAAGCGAACT	462 bp
Exon12.1	AGTTGGCATGATAGGAGCTCA	GGATGGAAACAAAAGAGGCTT	453 bp
Exon12.2	TGGGGTCAATGTTATGGACC	TCCTGGAAATTACCCCAAAG	335 bp
Exon13	GCATAATGATCTAGGCCATGG	CCTATAGGGCTGCACGGTAA	413 bp
Exon14	GCTGCTGTTAAGTTCCCATTACA	AAGCCAAAGAGAAGATGAGGC	397 bp
Exon15	GTTCATCCTACCATGCCACA	TGATTCAAAATCCTGTTGTCCA	413 bp
Exon16	TTTGGTTGTATACAGTTGATGGTGA	AGGCAAACCACAGCACAGTC	404 bp
Exon17	GTTTACCCAATGGGGTCATG	AGCAGCTGCCAAGACAAAAG	448 bp
Exon18	TCTTCTGCCAAGATGTGGTG	CAGGGGAGTACCTCGGAAA	358 bp
Exon19	CTACCCAGCAATCATTTGTGG	TGCTAATTTATTTCCTGAGCTTTTT	310 bp
Exon20	GTGCAAGGGCCTATCCTAGG	CCAAGTCACATCTGGTAGAACCA	304 bp
Exon21	ATGTGCAGTGAGTTTGCCAA	CGGCTGACTTTGCTAGAGTC	338 bp
Exon22	GTTTACCCAATGGGGTCATG	AGCAGCTGCCAAGACAAAAG	448 bp
Exon23.1	AGGTGGAATCAAAGCAGCCT	CACGCCTTCCTATGAAGTCC	414 bp
Exon23.2	AATCAGAATGCCTGTTTGTGG	TTCTTAGGCTTGTAAGCAATGAG	452 bp
3′UTR region	TCTCAATTTTATCCCTCACCTG	TAAAGTATAATAAGGACATGTGGCA	472 bp

bp: base pair.

3′UTR: 3′ untranslated region.

**Table 2:** Descriptions of patients with venous malformation.

Family	Patient symbol	Gender	Number of lesions	Location	Other patients in family
VMF01	VMF01.II.2	F	1	Lip	No
VMF02	VMF02.III.2	F	1	Lip	No
VMF03	VMF03.III.2	F	1	Lip	No
VMF04	VMF04.III.3	F	1	Lip	Yes
VMF04	VMF04.III.9	F	1	Lip	Yes
VMF05	VMF05.III.3	F	1	Lip	No
VMF06	VMF06.III.1	M	1	Lip	No
VMF07	VMF07.III.4	M	1	Genio-cervical	No
VMF08	VMF08.III.7	F	1	Lip	No
VMF09	VMF09.III.5	F	1	Lip	No
VMF10	VMF10.III.2	M	1	Lip	No

F: female; M: male.
